# Advance of Asiatic Cholera

**Published:** 1847-06

**Authors:** 


*Advance of Asiatic Cholera.—Great Mortality in Mecca.*—By intelligence received from Alexandria, dated January 19th, we learn that the Asiatic cholera has reappeared in the Hedjas, extending as far as Aden, and that within the space of a few days, more than fifteen thousand persons have been cut off by this terrible disease, in Mecca and its environs. It had, however, somewhat diminished in intensity, and was extending southwards. On the receipt of this intelligence great alarm was excited in Egypt, and a rumor soon spread that the disease had reached Suez and even Cairo. This report, however, was unfounded. The cholera had not reached Djedda on the 28th December. A sanatary cordon was about to be placed at a few leagues distance from Suez, so that no pilgrims would be allowed to enter Egypt from that quarter.—*Ib.*

